# Tailoring the Morphology and Electrochemical Behavior of TiO_2_ Powders via Surfactant-Assisted Synthesis by a Modified Pechini Route

**DOI:** 10.1021/acs.langmuir.6c01008

**Published:** 2026-05-04

**Authors:** Patrik Yuichi Aoyague, Edson Araujo de Almeida, Regiane da Silva Gonzalez, Jéssica de Lara Andrade, Nelson Consolin Filho, Osvaldo Valarini Junior, Ana Paula Peron, Gideã Taques Tractz

**Affiliations:** † 74354 Universidade Tecnológica Federal do Paraná, Chemistry Department, Rosalina Maria dos Santos Avenue, 87301-899 Campo Mourão, PR, Brazil; ‡ Universidade Estadual de Maringá, Chemistry Department, Colombo Avenue, 87020-900 Maringá, PR, Brazil

## Abstract

Searching for sustainable alternatives to environmental challenges has driven significant interest in metal oxides, particularly TiO_2_, due to its versatility in energy conversion, pollutant adsorption, and electrochemical applications. TiO_2_ powders were synthesized via a modified Pechini method, comparing the conventional route with the use of the Tween-80 surfactant as a structure-directing agent. Structural, morphological, and electrochemical characterizations were performed to assess the impact of the synthesis on material performance. Thermogravimetric analysis (TG) revealed distinct decomposition profiles, with the Tween-based precursor exhibiting enhanced thermal stability. X-ray diffraction confirmed the formation of anatase–rutile structures, with Tween-80 favoring a higher anatase content (74.3% anatase phase). Scanning electron microscopy (SEM) and specific surface area (*S*_BET_) measurements demonstrated that Tween-80 promoted a more porous and fragmented morphology, leading to a 21-fold increase in specific surface area. ζ potential and XRD–Rietveld analysis further elucidated the influence of the organic precursor on the surface charge and crystallite size. Electrochemical impedance spectroscopy (EIS) showed a significant reduction in charge transfer resistance (65.8 kΩ), compared to that of ethylene glycol-based films (943 kΩ), while cyclic voltammetry (CV) in the presence of methylene blue indicated improved redox activity, enhancing the applicability of this TiO_2_ powder in several areas.

## Graphical abstract

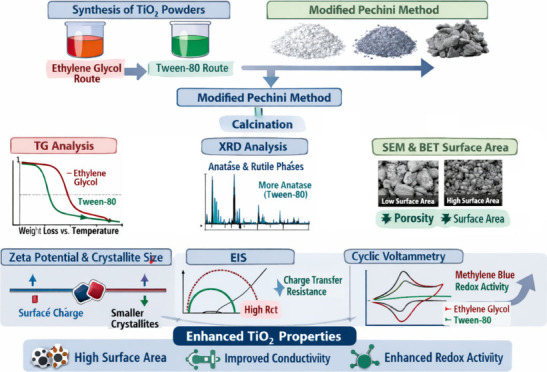

## Introduction

Environmental challenges such as pollution and climate change continue to impact populations worldwide, highlighting the urgent need for innovative and sustainable solutions.^1,2^ Among the emerging tools, nanotechnology has gained prominence for its potential to address these issues effectively.^3^ Despite its complex nature, it offers tangible pathways toward environmental remediation and energy efficiency, particularly when applied with a thoughtful design and purpose.

In this context, the use of semiconductor-based nanomaterials has attracted a significant amount of attention due to their versatility across various technological domains. Applications in solar energy conversion,^3^ modified electrodes,^4^ pollutant adsorption,^5^ and energy storage devices such as supercapacitors^6^ frequently depend on these materials, whose properties can be finely adjusted through structural and compositional modifications.

Oxides such as TiO_2_, Nb_2_O_5_, ZnO, and CeO_2_ are commonly synthesized using distinct methodologies,^7,8^ such as hydrothermal,^8,9^ coprecipitation,^10^ and Pechini routes.^11^ For instance, Guimaraes and co-workers reported the hydrothermal synthesis of TiO_2_ nanoparticles, obtaining powders composed of anatase and rutile phases.^8^ Although this method yields crystalline materials with desirable properties, its dependence on high-pressure and high-temperature reactors remains a drawback. In contrast, Chen and co-workers demonstrated the synthesis of CeO_2_ via coprecipitation, a technique that offers low-temperature processing as a key advantage, although this method displays limited flexibility in tuning the final characteristics of the materials.^10^ Interestingly, the Pechini method has emerged as a compelling alternative, offering better control over composition and homogeneity while operating under milder conditions.^12^

The Pechini method remains a widely employed route for the synthesis of metal oxides due to its simplicity, cost-effectiveness, and ability to produce homogeneous multicomponent systems.^11,12^ However, one of its well-documented limitations is the formation of extensive particle agglomerates, often attributed to the complexation and polymerization dynamics inherent in the process. In light of this, it is interesting to explore the substitution of traditional reagents, such as ethylene glycol or citric acid, with alternative organic compounds to mitigate aggregation and enhance morphological control and surface modifications.

The incorporation of surfactants, such as Tween-80, is particularly attractive due to their bulky hydrophilic head groups and amphiphilic structure. Rather than participating in the polyesterification reaction, Tween-80 acts as a supramolecular structuring agent during gel formation.^13,14^ When used above its critical micelle concentration, it promotes the formation of micellar domains that serve as transient soft templates, enabling spatial confinement of titanium species.^15–17^ This confinement can limit particle growth, reduce agglomeration, and ultimately influence the nanoparticle morphology and surface area.

In contrast to conventional low-molecular weight polyols such as ethylene glycol, which are directly involved in polyesterification, Tween-80 primarily modulates the organization of the reaction medium through noncovalent interactions.^13,14^ Its ability to induce micellar structuring introduces an additional level of control over nucleation and growth processes, analogous to soft-templating strategies widely reported in mesostructured materials.^15–17^ Despite the extensive use of surfactants in nanomaterial synthesis, their role as supramolecular organizers within polymeric precursor routes such as the Pechini method and the resulting impact on electrochemical behavior remain comparatively underexplored.

In this context, this study focuses on engineering TiO_2_ powders and films with improved interfacial charge transfer properties, aiming at electrochemical applications such as sensing and related redox-based systems.

## Experimental Section

TiO_2_ powders were synthesized by using the Pechini method. Initially, 35.0 mL of ethylene glycol (Vetec) or Tween-80 (Dinâmica), with molecular structures presented in Figure S1, was added to separate beakers under constant agitation. Subsequently, 9.6 g of citric acid (Vetec) was introduced into each beaker, and the mixture was heated to 70 °C until complete homogenization was achieved. Afterward, 5.0 mL of titanium isopropoxide (Aldrich) was added, and the temperature was increased to 100 °C. No additional water was intentionally added during the synthesis, as titanium isopropoxide is highly sensitive to hydrolysis and can readily react with ambient moisture as well as water generated during the polyesterification process.^18,19^ The resulting resin was first subjected to a brief heat treatment at 400 °C to partially decompose the organic matrix, promote particle deagglomeration, and enhance the surface area. This was followed by calcination at 600 °C for 3 h to achieve complete crystallization of TiO_2_ nanoparticles.

The chemical characterization of the resin produced was performed by using Fourier transform infrared spectroscopy (FTIR). Measurements were obtained with a dual-beam Fourier transform spectrophotometer (Carry 600 Series, Agilent Technologies) in the range from 4000 to 400 cm^–1^.

Thermogravimetric resin analyses were performed using a thermogravimetric analyzer (TGA-50, Shimadzu) in the temperature range from 25 to 800 °C. Synthesized powders were evaluated by XRD measurement, using D2 phase Bruker equipment, and ζ potentials were measured in Litesizer 500, Anton PAAR equipment (in an ethanol solvent). TiO_2_ particles were also analyzed by scanning electron microscopy, with magnifications of 300× and 5K×, in a Tescan Vegan Microscope and by transmission electron microscopy (JEOL-JEM 1400). The energy band gap was estimated by the Kubelka–Munk equation (eq 1 of the Supporting Information) using reflectance spectra obtained in a UV–vis–NIR PLUS Shimadzu spectrophotometer.^20^ The textural characteristics were determined by N_2_ physisorption using a surface area analyzer (Novatouch LX2 QuantaChrome) at 77 K. Specific areas (*S*_BET_) were calculated with QuantaChrome TouchWin using the Brunauer–Emmett–Teller (BET) equation^21,22^ in the relative pressure (*p*/*p*_0_) range of 0.050–0.300, in accordance with IUPAC recommendations.^22^

Films using synthesized oxides were produced as follows. First, 3 g of oxide was inserted into a mortar, followed by 0.1 mL of acetyl acetone, 0.1 mL of Triton-X, 3 mL of deionized water, and 0.1 mL of polyethylene glycol. The paste was macerated for 30 min, and the emulsion was coated onto the FTO (fluorine-doped tin oxide from Aldrich) substrate by a doctor blading methodology.^23^ Films were calcined for 30 min at 450 °C and evaluated by electrochemical impedance spectroscopy (EIS) (10 kHz to 10 mHz with an amplitude of 10 mV) and cyclic voltammetry (CV) (−1.5 to 1.0 V with a scan rate of 25 mV/s) in a 0.1 M KCl solution as the supporting electrolyte, using a three-electrode system with FTO/TiO_2_ as the working electrode, Pt as the counter electrode, and Ag/AgCl as the reference electrode. Electrochemical measurements were performed in a Metrohm potentiostat.

## Results and Discussion

### Analysis of the Resin

The resin produced by the modified Pechini method, using both Tween-80 and ethylene glycol, was analyzed by FTIR, and the spectra are depicted in Figure 1.

**Figure 1:**
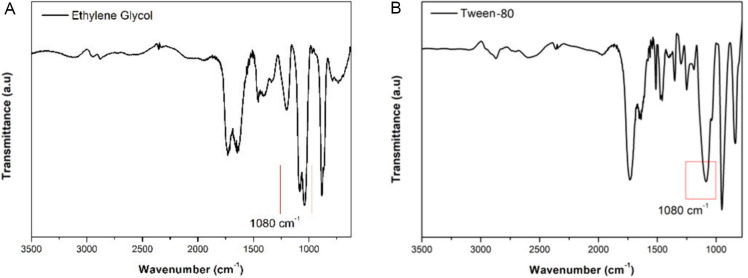
FTIR spectra of resins produced using ethylene glycol and Tween-80 as structuring agents.

The FTIR spectra presented in panels A and B of Figure 1 further show that both resins exhibit similar overall spectral features, with the presence of an intense band at approximately 1750 cm^–1^, attributed to the carbonyl (C=O) stretching of citric acid and ester groups formed during the polyesterification of the Pechini route. Additionally, the presence of bands in the low-frequency region (∼800 cm^–1^) suggests vibrations associated with Ti–O or Ti–O–C linkages, which supports the incorporation of titanium into the polymeric matrix.^24^

Marked differences are observed in the ∼1080 cm^–1^ region between the resins synthesized using ethylene glycol and Tween-80. For the ethylene glycol-based resin (Figure 1A), a clear band splitting is observed in this region, which is characteristic of the symmetric and asymmetric stretching vibrations of C–O–C linkages, indicating the formation of a more organized polyester network through esterification with citric acid. In contrast, the resin synthesized with Tween-80 exhibits only a broad band around ∼1080 cm^–1^, predominantly attributed to C–O stretching vibrations of the ethoxylated chains of the surfactant, suggesting a lower degree of esterification, likely due to the steric hindrance imposed by the bulky molecular structure.^24^

Thermogravimetric analysis (TG and DTG) of TiO_2_ resins obtained by the conventional and modified Pechini methods is shown in panels A and B, respectively, of Figure 2.

**Figure 2:**
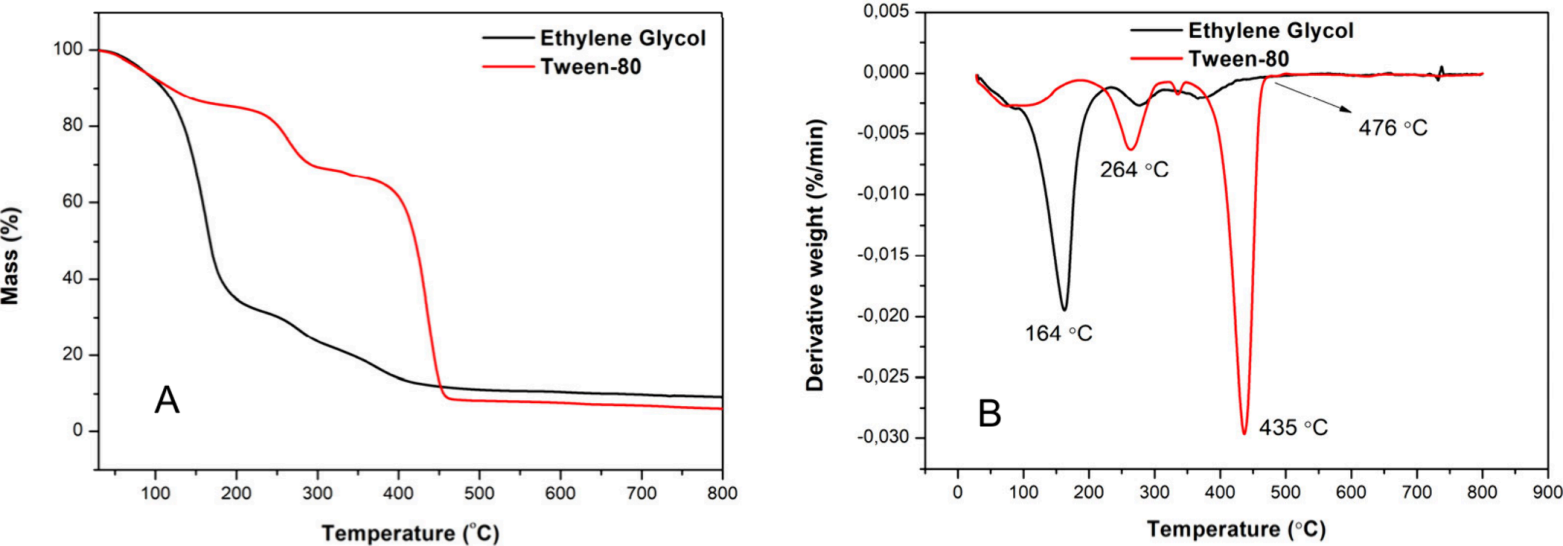
(A) TG and (B) DTG curves for the TiO_2_ resin produced by the traditional and modified Pechini route with surfactant assistance.

The TG and DTG curves reveal distinct decomposition behaviors for the TiO_2_ precursors synthesized with ethylene glycol and Tween-80, highlighting the influence of the organic agents on the thermal stability of the polymeric network formed during the Pechini process. The system synthesized with ethylene glycol exhibited an earlier decomposition profile, with a primary DTG peak at 164 °C, attributed to the evaporation and degradation of low-molecular weight species.^25,26^ Ethylene glycol, a small linear diol with two reactive hydroxyl groups, forms less stable complexes with citric acid and titanium isopropoxide. This structural simplicity leads to a faster and less controlled decomposition, followed by a minor event near 264 °C, with thermal stabilization occurring above 476 °C.

For the Tween-based route, two major mass loss events were observed, with peaks centered at 264 and 435 °C. The first peak can be attributed to the breakdown of less thermally stable segments, particularly polyoxyethylene chains (−(CH_2_–O–CH_2_)*_n_*) linked to the hydrophobic alkyl backbone of the surfactant. These parts are susceptible to oxidative cleavage, as previously reported.^27^ The second event, at 435 °C, corresponds to the combustion of residual organic matter and the final transition from an amorphous matrix to the inorganic TiO_2_ phase.^27^

The molecular architecture of Tween-80 features branched chains containing ester and ethoxylated groups and a long alkyl tail. This amphiphilic nature promotes supramolecular organization in solution and leads to the formation of denser and more thermally stable polymeric networks, which may even form micellar structures. The increased decomposition temperature observed is consistent with such structural complexity.^27,28^

### Structural and Morphological Analysis

Therefore, to evaluate the impact of organic matrices in phase formation, XRD analysis was performed, and the results are depicted in Figure 3.

**Figure 3:**
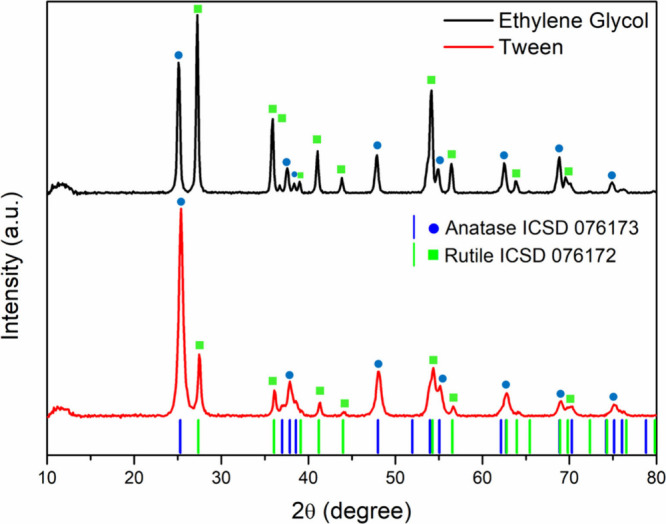
XRD patterns of TiO_2_ produced by the Pechini methodology with ethylene glycol and Tween-80 as alcohol precursors.

Figure 3 shows that both synthesized TiO_2_ samples presented two crystalline phases: anatase and rutile. Qualitative analysis of Rietveld refinement (Figure S2) allowed for the estimation of the relative content of each phase. Particles produced with ethylene glycol as a modifying agent presented 43.5% anatase phase and 56.5% rutile phase, while those synthesized with Tween-80 presented 74.3% anatase phase and 25.7% rutile phase. These results indicate that the choice of the structure-directing agent directly influences the proportion of crystalline phases formed. Specifically, the use of smaller and simpler molecules favored the majority formation of the rutile phase, while the use of a larger and more complex molecule promoted the predominance of the anatase phase. This behavior may be related to steric effects, interference from the reaction medium, or the differentiated interaction of agents with metal precursors during polymerization in the Pechini route.

The Rietveld refinement shown in the Supporting Information demonstrates excellent agreement between the experimental data and the X-ray diffraction patterns derived from ICSD crystallographic files 076173 and 076172. As observed, both TiO_2_ phases, anatase and rutile, exhibit a tetragonal crystal system. The anatase phase is associated with space group *I*4_1_/*amd* (141), whereas the rutile phase corresponds to space group *P*4_2_/*mnm* (136).^29^

The literature has already demonstrated that parameters such as the concentration of metal precursors significantly influence the ratio between the TiO_2_ phases.^30^ Similarly, the data obtained in this study suggested that the modification of the alcohol agent can be a decisive factor in the stabilization of one crystallographic phase over the other, thus representing a relevant variable for the structural control of the material. Table 1 summarizes the lattice parameters, unit cell volumes, densities, and average crystallite sizes, as determined from the refinement.

**Table 1:** Results of Rietveld Refinement by XRD of Titanium Oxide (TiO_2_) Samples Produced Using Ethylene Glycol and Tween-80

anatase (ICSD 076173)					rutile (ICSD 076172)				
tetragonal, space group *I*4_1_/*amd* (141), α = β = γ = 90°					tetragonal, space group *P*4_2_/*mnm* (136), α = β = γ = 90°				
*a* (Å)	*b* (Å)	*c* (Å)	*V* (Å)	ρ (g/cm^3^)	*a* (Å)	*b* (Å)	*c* (Å)	*V* (Å)	ρ (g/cm^3^)
3.777^a^	3.777^a^	9.501^a^	135.54^a^	3.92^a^	4.584^a^	4.584^a^	2.953^a^	65.02^a^	4.28^a^
3.783^b^	3.783^b^	9.484^b^	135.83^b^	3.90^b^	4.597^b^	4.597^b^	2.955^b^	62.45^b^	4.29^b^
3.803^c^	3.803^c^	9.548^c^	138.07^c^	3.842^c^	4.608^c^	4.608^c^	2.973^c^	63.15^c^	4.21^c^
Ethylene crystalline size, 26.96 ± 2.62 nm					Ethylene crystalline size, 27.59 ± 3.56 nm				
Tween crystalline size, 17.39 ± 3.12 nm					Tween crystalline size, 18.73 ± 2.77 nm				

a Values obtained from crystallographic data sheets ICSD 076173 and ICSD 076172.

b Values obtained experimentally using crystallographic data sheets ICSD 076173 and ICSD 076172, for samples prepared with Tween.

c Values obtained experimentally using crystallographic data sheets ICSD 076173 and ICSD 076172, for samples prepared with ethylene glycol.

As shown in Table 1, lattice parameters *a*–*c*, as well as the unit cell volumes and densities, remain closely aligned with the values reported in the corresponding crystallographic files, indicating good structural fidelity of the synthesized material. However, a reduction in the average crystallite size is observed in the samples synthesized with Tween-80 compared with those prepared using ethylene glycol. This result suggests that the molecular size of the structure-directing agent may influence crystal growth, potentially due to steric hindrance or changes in the nucleation rate during the polymeric gel formation step of the Pechini route.

To verify the particle morphology, the micrographs were obtained by scanning electron microscopy and transmission electron microscopy and are shown in Figure 4A–D.

**Figure 4:**
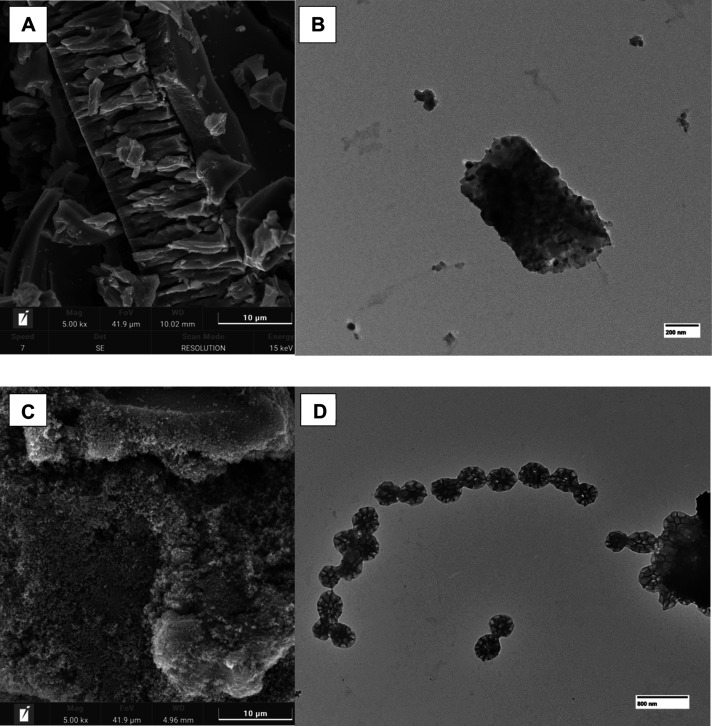
(A and C) SEM images and (B and D) TEM images of TiO_2_ powders. Panels A and C show SEM images of powders produced with Ethylene glycol and Tween-80, respectively (5K× magnification), while panels B and D show TEM images of the same samples at the corresponding magnifications.

The micrographs highlight clear differences in the morphologies of the TiO_2_ powders depending on the precursor used. Samples synthesized with ethylene glycol displayed a compact surface at the microscale. This smoother texture likely results from a more extensive polymeric network formed during resin formation, leading to a compact structure. Such features are usually associated with a reduced surface area, which may be a drawback for applications requiring high interfacial dynamics.^31^

In contrast, when Tween-80 was used, the morphologies of the surface appeared to be markedly different. At 5K× magnification (Figure 4C), the material showed a fragmented, loosely rough bound structure, with particles scattered. This morphology resembles a powdery texture, suggesting a greater distance between particles in the gel matrix.^32^ The surfactant likely interferes with cross-linking, through either steric effects or alteration of the local environment during polymer formation. Furthermore, its amphiphilic character may contribute to micellar arrangements during synthesis, enhancing porosity and irregularity, as observed in the TEM image of Figure 4D.^33^

Regarding the mechanism of particle formation, based on the proposed mechanism, the formation of TiO_2_ nanoparticles in the conventional Pechini method is governed by a sequence of esterification, complexation, and thermal decomposition steps. Initially, the carboxylic groups of citric acid react with ethylene glycol via polyesterification, leading to the formation of a three-dimensional polymeric network and the concomitant release of water molecules. This polymeric matrix plays a crucial role in homogeneously dispersing the metallic species at the molecular level.^34^

Simultaneously, the titanium precursor, titanium isopropoxide, undergoes partial hydrolysis due to its high sensitivity to ambient moisture and to water generated in situ during polyesterification. The resulting Ti^4+^ species interact strongly with citrate ligands through coordination involving carboxylate groups, forming stable chelated complexes. These interactions ensure a uniform distribution of Ti ion species within the organic resin.^35,36^

Upon calcination, the organic matrix is thermally decomposed, promoting the condensation of Ti–O–Ti bonds and leading to the formation of crystalline titanium dioxide nanoparticles.^37^ In contrast, in the modified Pechini route assisted by surfactant Tween-80, an additional level of structural organization is proposed. No covalent reaction is expected between citric acid and Tween-80, as ether groups are not reactive toward carboxylic acids under the employed conditions (as also discussed in Figure 1B). Instead, their interaction is suggested to be dominated by weak intermolecular forces, such as van der Waals interactions and hydrogen bonding.^13,14^

When Tween-80 is used above its critical micelle concentration, the system is proposed to favor the formation of micellar aggregates, as suggested by the TEM images of Figure 4D. In this environment, citric acid molecules are distributed around or within the hydrophilic regions of the micelles, promoting increased spacing between surfactant head groups. This effect can be interpreted as a micelle expansion phenomenon, analogous to the role of swelling agents in mesostructured silica systems.^15–17^ Although no long-range mesostructural ordering is expected, the role of surfactant micelles as transient soft templates resembles the mechanisms reported for mesoporous silica systems and is described in a schematic overview in Figure S3.^15,17^

Upon addition, partially hydrolyzed Ti^4+^ species are proposed to preferentially coordinate with citrate ligands rather than interact with the ether functionalities of Tween-80. This preference arises from the stronger Lewis basicity and potential bidentate coordination of carboxylate groups, which confer enhanced stability through the chelate effect and stronger metal–oxygen interactions compared to neutral ether oxygen atoms.^35,36^ Therefore, no significant displacement of the citrate ligands by Tween-80 is expected.

Additionally, Tween-80 is proposed to adsorb at the interface of the forming inorganic–organic microphases. In this configuration, the hydrophilic polyoxyethylene chains are oriented toward the polar medium, while the hydrophobic alkyl chain extends toward less polar regions. This arrangement may act as a steric barrier, reducing particle coalescence and influencing particle growth.

The adsorption of Tween-80 at the interface of the forming inorganic–organic microphases is consistent with classical surfactant self-assembly behavior, in which amphiphilic molecules minimize the interfacial free energy by orienting their hydrophilic polyoxyethylene chains toward polar environments and their hydrophobic moieties toward less polar domains.^38,39^ In this context, Tween-80 does not compete with citrate ligands for coordination with Ti^4+^ species, as ether groups exhibit significantly lower affinity for metal centers compared to carboxylate groups.^35,36^ Instead, the surfactant modulates the local chemical environment in which chelation occurs, providing spatial confinement and steric stabilization without disrupting the metal–citrate complexation equilibrium.

This behavior suggests that Tween-80 primarily influences particle formation through interfacial organization and confinement effects rather than through direct chemical interaction with the titanium precursor. Furthermore, nonhydrolyzed alkoxide species may partition into the hydrophobic core of the micelles, creating distinct microenvironments where hydrolysis and condensation reactions can occur in a spatially confined manner. This dual distribution, between the micellar interface and core, may contribute to heterogeneous nucleation pathways.^38,39^

During calcination, the removal of the organic matrix and surfactant leads to the collapse of these structured domains, generating TiO_2_ nanoparticles with increased interparticle spacing and an enhanced surface area. The expanded micellar structures, modulated by citric acid, are proposed to act as transient templates that ultimately influence the morphological and textural properties of the final oxide material.^37^ Therefore, the role of Tween-80 is better described as a supramolecular regulator of nucleation and growth rather than a chemically reactive component of the Pechini network.

### Surface Area, Porosity, and Particle Stability

Overall, these observations indicate that using the Tween-80-assisted route instead of the ethylene glycol-assisted route in the Pechini method leads to a more open and less agglomerated structure. This change may provide a larger surface area, which is beneficial for surface-sensitive applications. In fact, N_2_ physisorption revealed significant differences in the specific surface area (*S*_BET_) between the two synthesis routes. The TiO_2_ synthesized via the standard Pechini method using ethylene glycol exhibited a relatively small surface area of 1.22 m^2^/g, consistent with the more agglomerated and compact morphology observed in the micrographs. In contrast, the TiO_2_ obtained with Tween-80 showed a substantially larger surface area of 26.10 m^2^/g, which is in accordance with the previously described micrographs as an open and fragmented structure (Figure 4C,D).

For comparison, Heydari et al.^40^ reported a specific surface area of approximately 17 m^2^/g for black TiO_2_ calcined at 400 °C under a controlled argon atmosphere. Although this value is within the range typically observed for nanostructured TiO_2_, it is important to note that such conditions involve controlled atmospheres, which differ from the synthesis approach employed in this study. Furthermore, the Pechini method is generally associated with the formation of materials with relatively small specific surface areas due to particle growth and agglomeration during calcination. This behavior has been reported in studies by Amaral et al.^41^ that describe oxide systems synthesized via the Pechini route exhibiting limited surface areas. This remarkable increase in surface area is probably associated with the interaction between the alkyl chain of the Ti precursor and the nonpolar tail of Tween-80. TEM images further confirm the reduced agglomeration and increased interparticle spacing in the Tween-based samples, corroborating the SEM observations.

Similarly, the steric repulsion among the polar head groups of Tween-80 may have kept the complexation spheres farther apart during the synthesis, leading to the formation of a more dispersed material with a significantly enhanced surface area, with enhanced interparticle porosity and particle fragmentation that are advantageous for applications relying on a high degree of surface exposure.^42^

In addition, ζ potential measurements, depicted in Figure S4, revealed that both samples exhibited negative values starting from pH 4.0, indicating a negatively charged surface under moderately acidic to basic conditions. The point of zero charge (PZC) was determined to be at pH 2.41 for the sample synthesized with ethylene glycol and pH 4.12 for the Tween-based sample, with good agreement with previously reported studies.^43^ Interestingly, although the material synthesized with ethylene glycol presented a more agglomerated morphology, it exhibited more negative ζ potential values across the pH range when compared with the TiO_2_ synthesized using the modified Pechini method. This behavior can be attributed to differences in surface chemistry and the nature of residual functional groups resulting from the synthesis route.^44^ The larger magnitude of the negative ζ potential in the ethylene glycol-derived sample suggests the presence of a greater density of defects on the surface, or stronger interaction with the ionic medium, which enhances its electrostatic repulsion despite the agglomerated state.^44^ This behavior can also be attributed to enhanced intercrystallization and sintering during calcination, which is further supported by the higher rutile phase content as observed in XRD analysis. These structural rearrangements likely dominate over electrostatic repulsion effects, leading to more compact agglomerates.^45^

In addition, the negatively charged surface of the material synthesized with ethylene glycol also makes it a promising candidate for the adsorption of cationic pollutants. Electrostatic attraction between the negatively charged surface and positively charged contaminants—such as methylene blue, crystal violet, and malachite green—can facilitate efficient removal from aqueous media. This characteristic highlights the potential of this material for environmental applications involving dye removal and water treatment.^5,46,47^

Regarding the electronic properties of the produced oxides, the band gap energies (*E*_gap_) were found to be 3.14 eV for the material synthesized via the conventional route using ethylene glycol and 3.20 eV for the TiO_2_ obtained with Tween-80, as shown in Figure S5. The slight blue-shift observed for the latter may be attributed to a greater proportion of the anatase phase, which is consistent with literature reports for this crystalline form.^9^ These differences in *E*_gap_ values suggest distinct redox capabilities, which could significantly influence their performance in photoelectrochemical applications. In particular, shifts in the conduction or valence band edges are expected, potentially altering the ability of the materials to facilitate redox reactions.^48^ To better understand the impact of these oxides on electronic behavior, the following section focuses on the fabrication and analysis of films prepared by using these materials.

### Electrochemical Performance

Figure 5 depicts the electrochemical impedance spectroscopy diagram (Nyquist plots) of TiO_2_.

**Figure 5:**
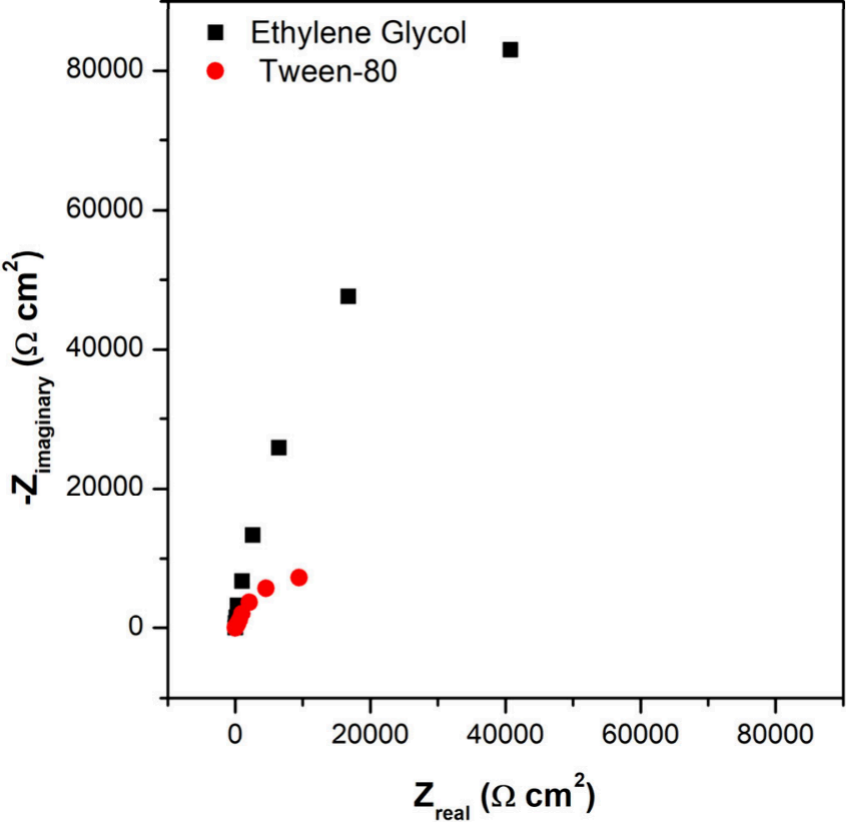
Nyquist diagram of TiO_2_ films produced with Ethylene glycol and Tween-80 under the FTO surface with 0.1 M KCl as the supporting electrolyte.

Figure 5 illustrates the electrochemical impacts of incorporating Tween-80 as a structure-directing agent during the synthesis of TiO_2_ films. Compared to conventional synthesis using ethylene glycol, the use of Tween-80 results in a more homogeneous and continuous film at the mesoscale while preserving higher scale porosity as evidenced by the micrographs in Figure 4. This enhanced uniformity is indicative of improved film integrity and is favorable for the formation of a more stable electric double layer (EDL).^48^ A denser EDL architecture is expected to facilitate more effective charge separation and accelerate interfacial electron transfer processes, thereby facilitating more effective charge separation, enhancing interfacial electron transfer, and improving ion transport.^49^

Electrochemical impedance spectroscopy (EIS) further substantiates these morphological observations. The Nyquist plots (Figure 5) reveal a clear enhancement in the capacitive behavior and a pronounced decrease in the interfacial resistance when Tween-80 is employed. Fitting the impedance data with a Randles equivalent circuit model (Figures S6 and S7) highlights this difference quantitatively. The charge transfer resistance (*R*_ct_) for TiO_2_ films synthesized with ethylene glycol was measured to be 943 kΩ, while films prepared using Tween-80 exhibited a substantially lower *R*_ct_ of 65.8 kΩ.^50^ This significant reduction in interfacial resistance is an important indicator of improved interfacial charge transfer dynamics, which is pivotal for devices relying on fast redox kinetics.^6,51,52^

These results indicate the critical role of surfactant selection in modulating the physicochemical properties of nanostructured metal oxides. The use of Tween-80 not only influences structure, particle packing, and porosity at the mesoscale but also directly enhances the electronic pathways and charge storage behavior of the material. Such findings pave the way for the rational design of next-generation functional films tailored for high-performance energy conversion and storage applications.

Comparisons with literature reports on commercial TiO_2_ systems indicate that charge transfer resistance and redox activity are strongly governed by factors such as phase composition, crystallite size, and specific surface area. As these parameters differ significantly from those obtained for the materials synthesized in this study, direct comparisons are not straightforward.^53^ To assess the electrochemical behavior resulting from the newly proposed synthesis route, the TiO_2_-based films were subjected to cyclic voltammetry (Figure 6) in the presence of methylene blue as a redox-active probe. This experimental approach aimed to evaluate the film’s potential applicability in sensing and analytical detection of specific analytes.

**Figure 6:**
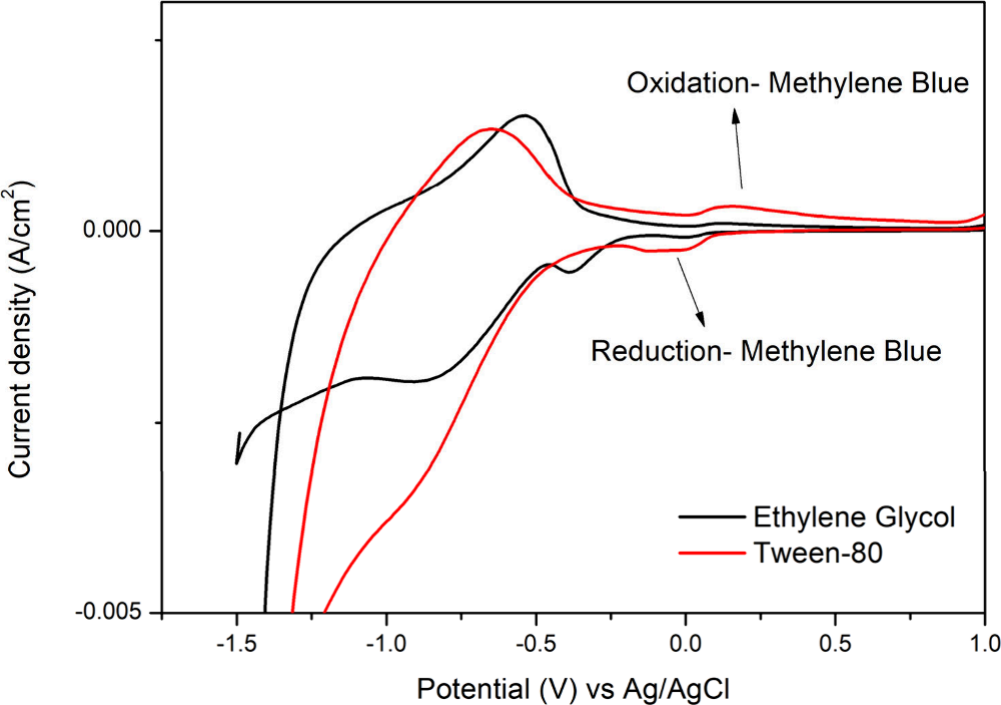
Cyclic voltammogram of TiO_2_ films produced with Ethylene glycol and Tween-80 in the presence of a methylene blue dye solution.

The voltammograms (Figure 6) obtained for the TiO_2_ films prepared with ethylene glycol and Tween-80 revealed notable differences in their electrochemical responses to methylene blue. The film synthesized via the new route exhibited a clear and distinct oxidation peak at 0.25 V versus the Ag/AgCl reference electrode, followed by a corresponding reduction peak, indicating efficient redox cycling of methylene blue.^54^ These peaks were sharp and well-defined, suggesting that the film surface properties and morphology favor fast and reversible electron transfer between the methylene blue molecules and the electrode surface.^55^

In contrast, the TiO_2_ film synthesized with ethylene glycol showed a much less significant response to methylene blue, with no clear oxidation or reduction peaks or significantly lower current densities. This result suggests that the film produced with ethylene glycol does not facilitate the oxidation or reduction of methylene blue to the same extent as the film produced with Tween-80.^56^ The absence of a clear redox peak can be attributed to the more compact, smoother morphology of the ethylene glycol-based film, which likely reduces the available surface area for effective interaction with methylene blue molecules.

Moreover, the denser packing of particles in the ethylene glycol-based film could hinder the diffusion of methylene blue to the electrode surface, limiting its electrochemical activity.

The enhanced oxidation and reduction behavior observed in the Tween-prepared film can be attributed to its unique morphology, as evidenced by the SEM and TEM images. The use of Tween during synthesis promotes an open, porous structure with fewer particle agglomerations, increasing the surface area available for interactions with methylene blue. Additionally, the surfactant–template morphology may help form a more uniform electric double layer (EDL) at the electrode interface, enhancing the film’s ability to store and transfer charge, thereby improving its electrochemical performance. The literature has also demonstrated the use of TiO_2_ to detect methylene blue dye by a self-doping process using cathodic polarization of 30 V during 24 h.^57^ From this perspective, the new route described in this paper provides an easier methodology to produce oxides and detect analytes in sensor production.^57^

Furthermore, the EIS results, which showed a significantly lower charge transfer resistance (*R*_ct_) (65.8 kΩ) for the Tween-based TiO_2_ film compared to the ethylene glycol-based film (943 kΩ), further support these findings. The lower *R*_ct_ suggests that the film synthesized with Tween-80 has a more efficient charge transfer pathway, which is critical for the observed enhanced redox response in the voltammetry experiments.^58^ Therefore, the electrochemical performance of TiO_2_ is influenced by multiple interrelated factors, including the phase composition, crystallite size, and specific surface area. In this study, the improved behavior observed for the Tween-80-derived material cannot be attributed to a single parameter. Instead, it results from a synergistic combination of structural and morphological features.^2^

These results highlight the importance of the film morphology and structure and the role of surfactants such as Tween-80 in modulating the electrochemical properties of TiO_2_ oxides. By improving the surface area and charge transfer dynamics, the Tween-based TiO_2_ film appears to be a more efficient platform for redox processes, making it a promising candidate for applications in sensors, energy storage devices, and other electrochemical systems in which fast and reversible electron transfer is crucial.

## Conclusions

The present study demonstrates that the incorporation of Tween-80 as a structure-directing agent in the Pechini synthesis route represents an effective strategy for modulating the structural, morphological, and electrochemical properties of TiO_2_ powders and films. While both synthesis routes produced crystalline TiO_2_ composed of anatase and rutile phases, the use of Tween-80 significantly favored the stabilization of the anatase phase and promoted a marked reduction in the crystallite size compared to the conventional ethylene glycol-based route.

Spectroscopic and thermal analyses revealed that the bulky and amphiphilic molecular structure of Tween-80 alters the polyesterification process and enhances the thermal stability of the polymeric resin, leading to a distinct decomposition behavior and influencing phase formation during calcination. These effects translated into pronounced morphological differences, with Tween-based TiO_2_ exhibiting a more open, less agglomerated architecture and a substantially larger specific surface area.

When they were processed into thin films, these structural advantages directly impacted the electrochemical performance. TiO_2_ films synthesized with Tween-80 displayed significantly lower charge transfer resistance and enhanced capacitive behavior, indicating improved interfacial charge transfer dynamics. Furthermore, cyclic voltammetry measurements using methylene blue as a redox probe demonstrated superior electrochemical activity for the Tween-derived films, highlighting their ability to facilitate fast and reversible electron transfer processes.

Overall, this work establishes that modifying the organic matrix in the Pechini method through the use of surfactants such as Tween-80 provides an accessible and versatile route for tailoring TiO_2_ nanostructures and films. The resulting improvements in surface area, phase composition, and interfacial electrochemical behavior make these materials promising candidates for applications in electrochemical sensing, energy conversion, and related redox-based systems.

## Supplementary Material



## Data Availability

The data that support the findings of this study are openly available on request from the corresponding author.

## Notes

Molecular structures of ethylene glycol and Tween-80, XRD diffractograms with Rietveld refinement, a schematic mechanism of TiO_2_ particle formation via the modified Pechini method, ζ potential measurements as a function of pH, band gap determination using Tauc plots based on the Kubelka–Munk function, and fitted EIS diagrams with their respective equivalent circuits for TiO_2_ films prepared with ethylene glycol and Tween-80 (PDF)
