# Supplementary material for: Tailoring the Morphology and Electrochemical Behavior of TiO2 Powders via Surfactant-Assisted Synthesis by a Modified Pechini Route
Source: Langmuir. 2026 May 4;42(19):13768–77. doi: 10.1021/acs.langmuir.6c01008 (PMC13192306; doi:10.1021/acs.langmuir.6c01008)
Supplement: Supplementary file 1 [file la6c01008_si_001.pdf]

TAILORING THE MORPHOLOGY AND ELECTROCHEMICAL BEHAVIOR OF TiO_2 POWDERS VIA SURFACTANT-ASSISTED SYNTHESIS BY MODIFIED PECHINI ROUTE

Patrik Yuichi Aoyague[a], Edson Araujo de Almeida[b], Regiane da Silva Gonzalez[a], Jéssica de Lara Andrade[a], Nelson Consolin Filho[a], Osvaldo Valarini Junior[a] Ana Paula Peron[a] and Gideã Taques Tractz[a*]

[a] Universidade Tecnológica Federal do Paraná, Chemistry Departament, Rosalina Maria dos Santos Avenue, 87301-899, Campo Mourão-PR, Brazil.

[b] Universidade Estadual de Maringá, Chemistry Department, Colombo Avenue,87020-900, Maringá-PR, Brazil

*gideatractz@utfpr.edu.br

https://orcid.org/0000-0002-4745-657X

SUPPORTING INFORMATION

CONTENTS:

Figure S1.A-B. Molecular structure of ethylene glycol in A and Tween 80 in B

Figure S2.A-B. XRD diffractograms of TiO_2 (ethylene glycol in A and Tween-80 in B) samples obtained experimentally and calculated using the Rietveld refinement method.

Figure S3. Mechanism of TiO_2 particles formation via modified Pechini method with Tween-80.

Figure S4. Zeta potential vs pH values to TiO_2 powders in suspension, produced with Tween-80 and Ethylene glycol by modified Pechini route.

Figure S5. Egap extrapolation in the Tauc plot from the Kubelka Munk Function to TiO2 produced by Pechini methodology with Ethylene glycol and Tween-80

Figure S6.A-B. Fitted EIS Diagram in A and equivalent circuit in B to TiO_2 film under FTO produced using ethylene glycol.

Figure S7.A-B. Fitted EIS Diagram in A and equivalent circuit in B to TiO_2 film under FTO produced using Tween-80.

$$F\,(R) = \frac{(1-R)^2}{2R} \text{(Equation 1)}$$

With R representing the Reflectance. This approach allows for the estimation of the band gap energy by plotting $[F(R)h\nu]^n$ versus photon energy ($h\nu$), where n is 1/2 for indirect transitions and 2 for direct transitions

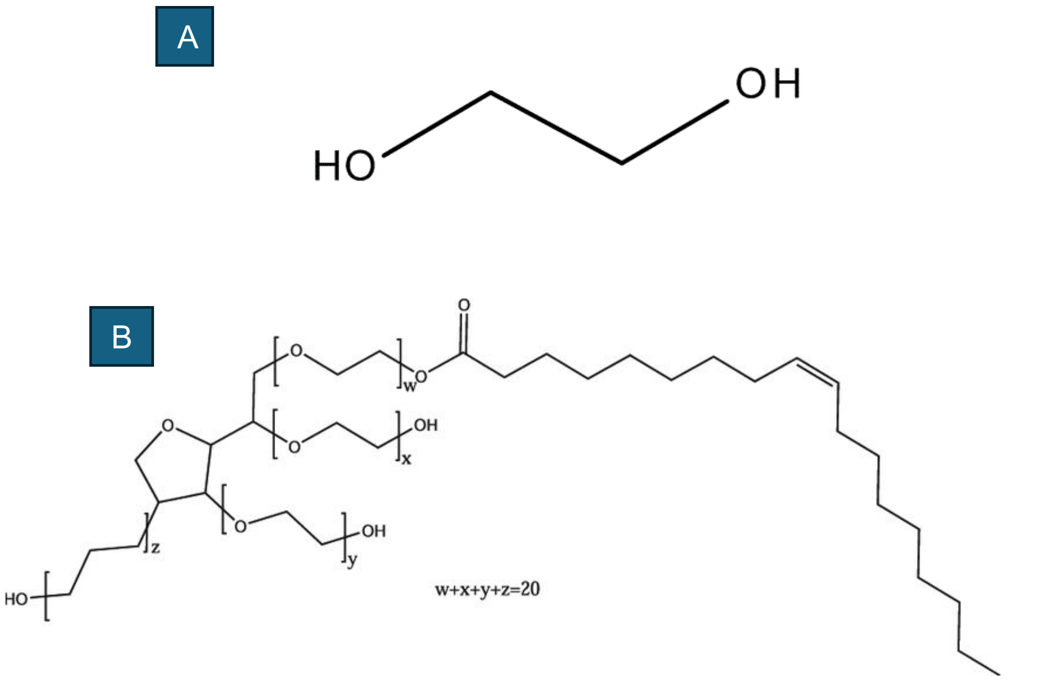

Figure 1.A-B SI. Molecular structure of ethylene glycol in A and Tween 80 in B

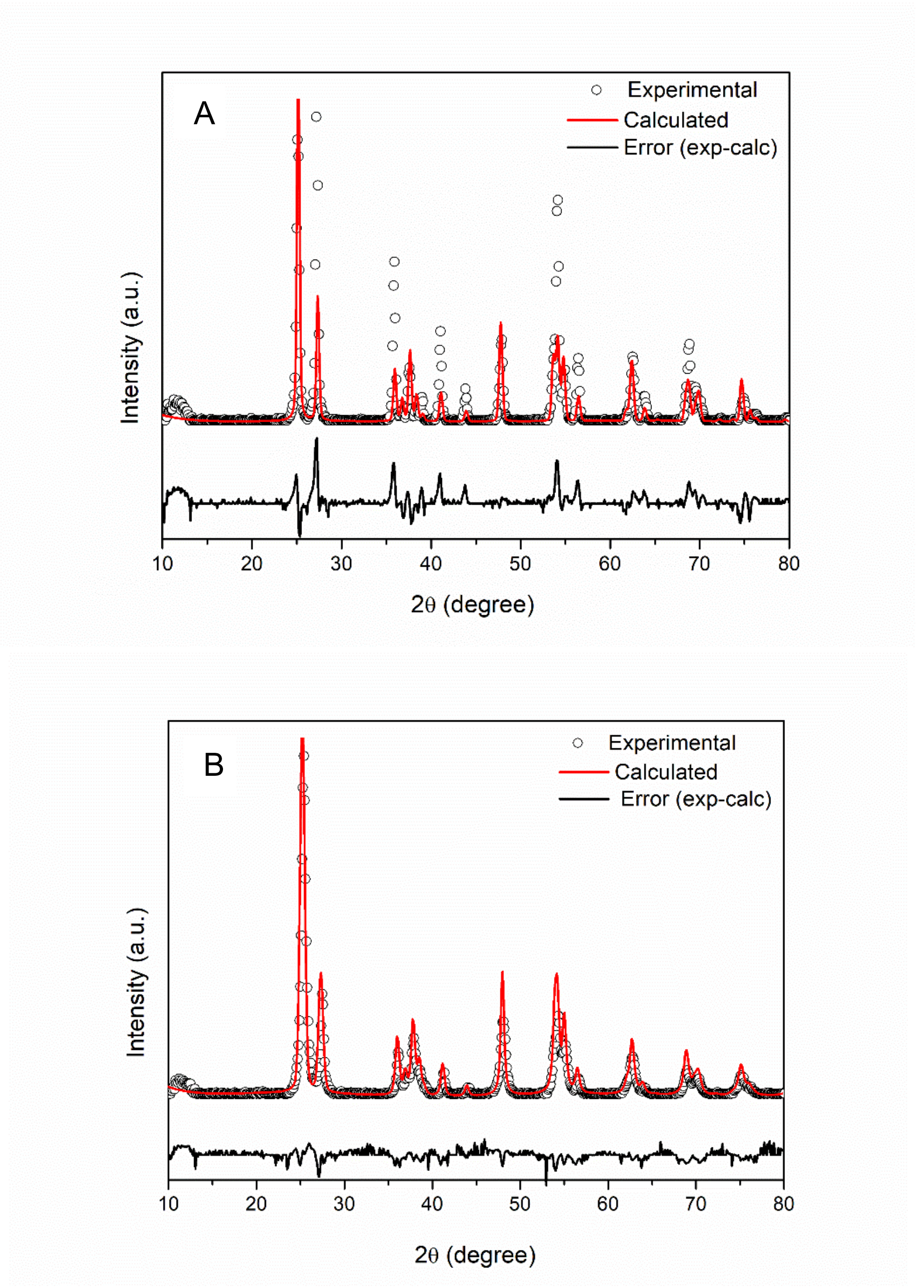

Figure 2A-B. SI XRD diffractograms of TiO_2 (ethylene glycol in A and Tween-80 in B) samples obtained experimentally and calculated using the Rietveld refinement method.

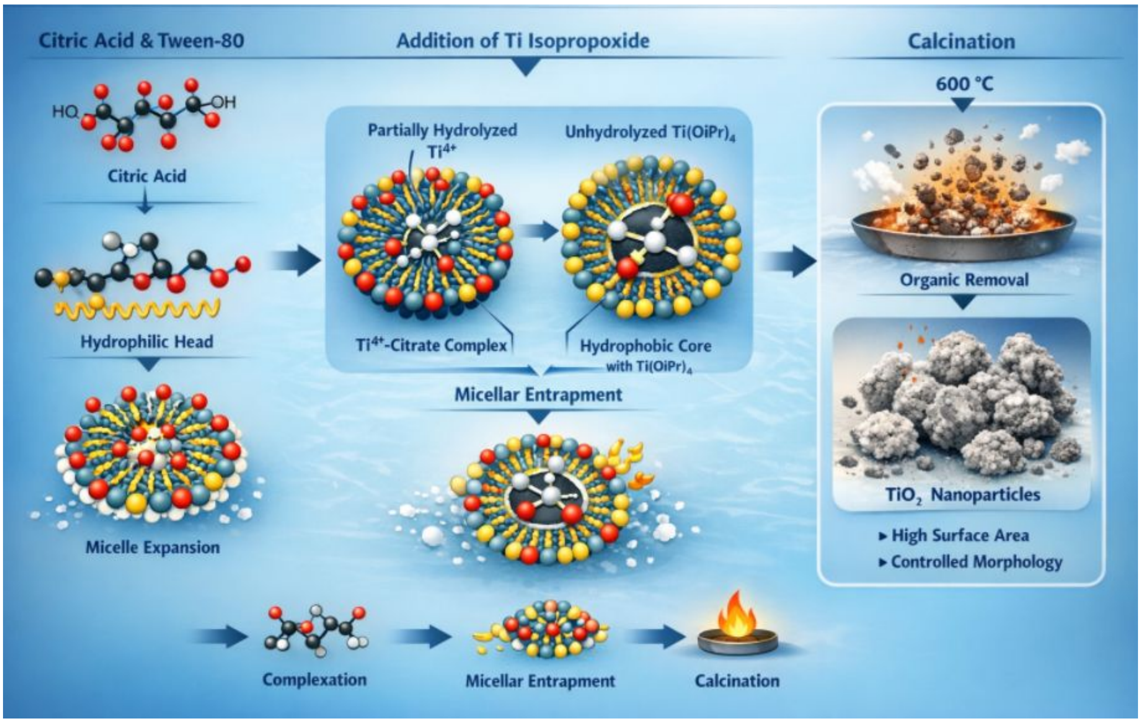

Figure 3. SI. Mechanism of TiO_2 particles formation via modified Pechini method with Tween-80

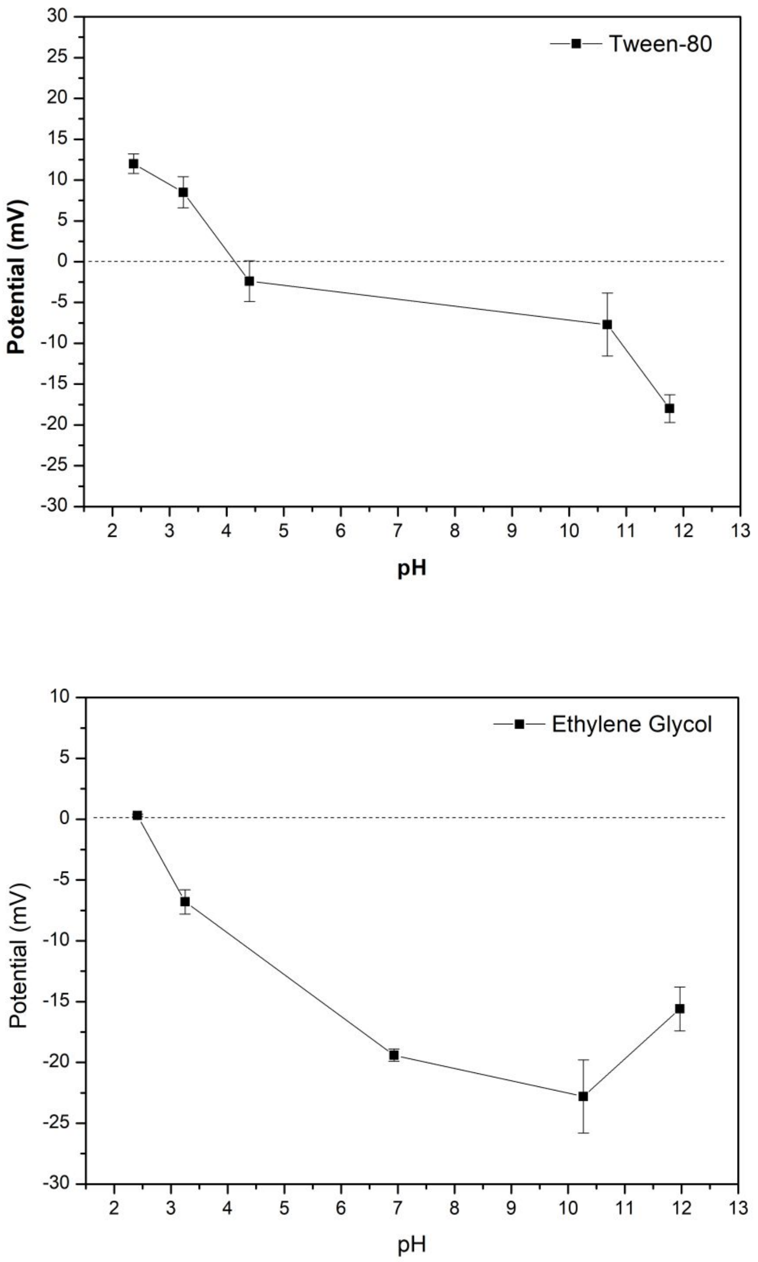

Figure 4.SI Zeta potential vs pH values to TiO_2 powders in suspension, produced with Tween-80 and Ethylene glycol by modified Pechini route.

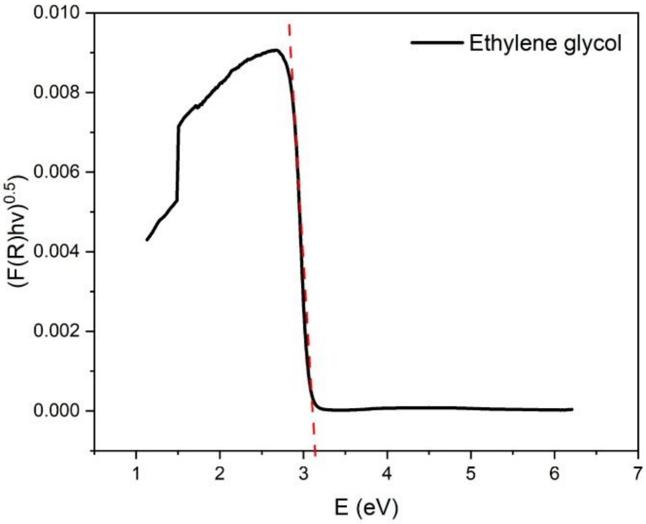

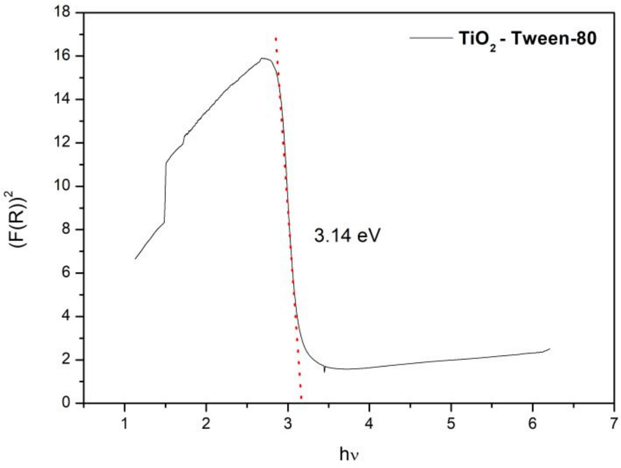

Figure 5.SI E_{gap} extrapolation in the Tauc plot from the Kubelka Munk Function to TiO_2 produced by Pechini methodology with Ethylene glycol and Tween-80

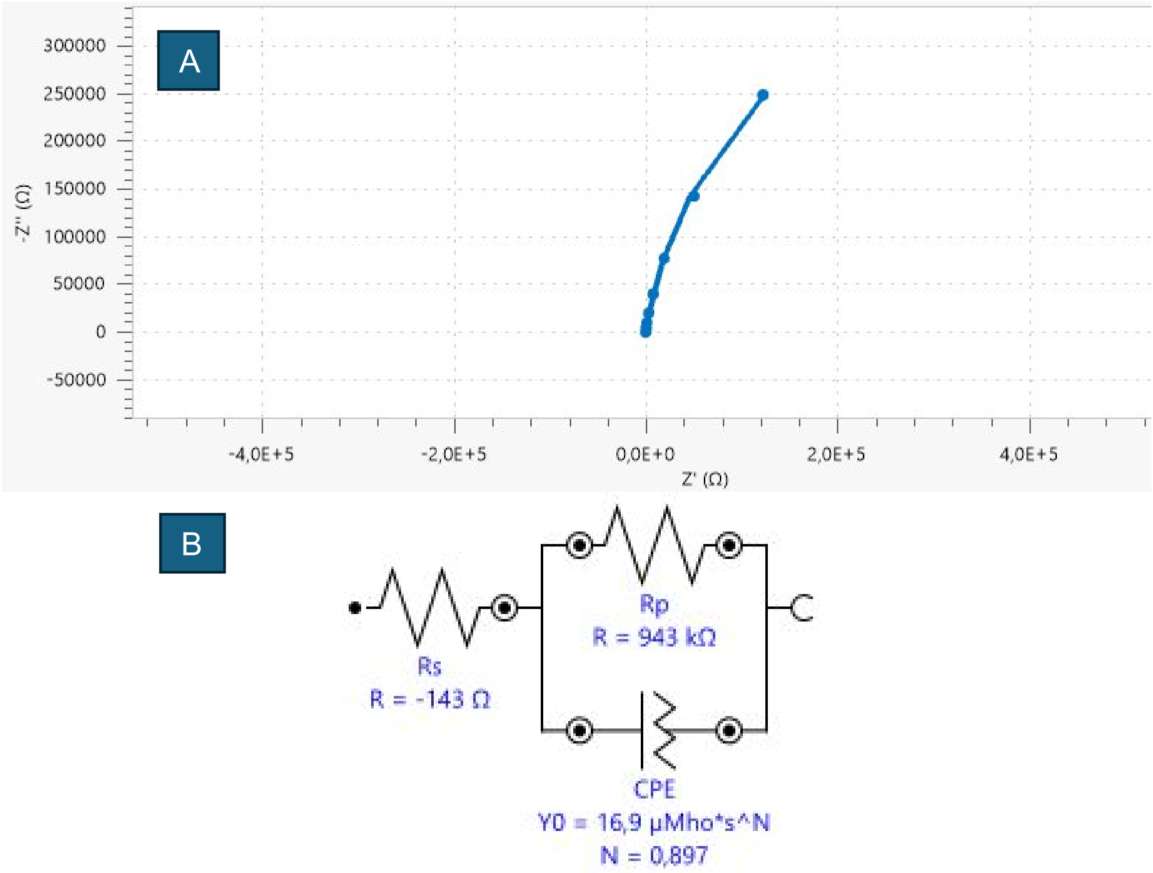

Figure 6A-B.SI Fitted EIS Diagram in A and equivalent circuit in B to TiO_2 film under FTO produced using ethylene glycol.

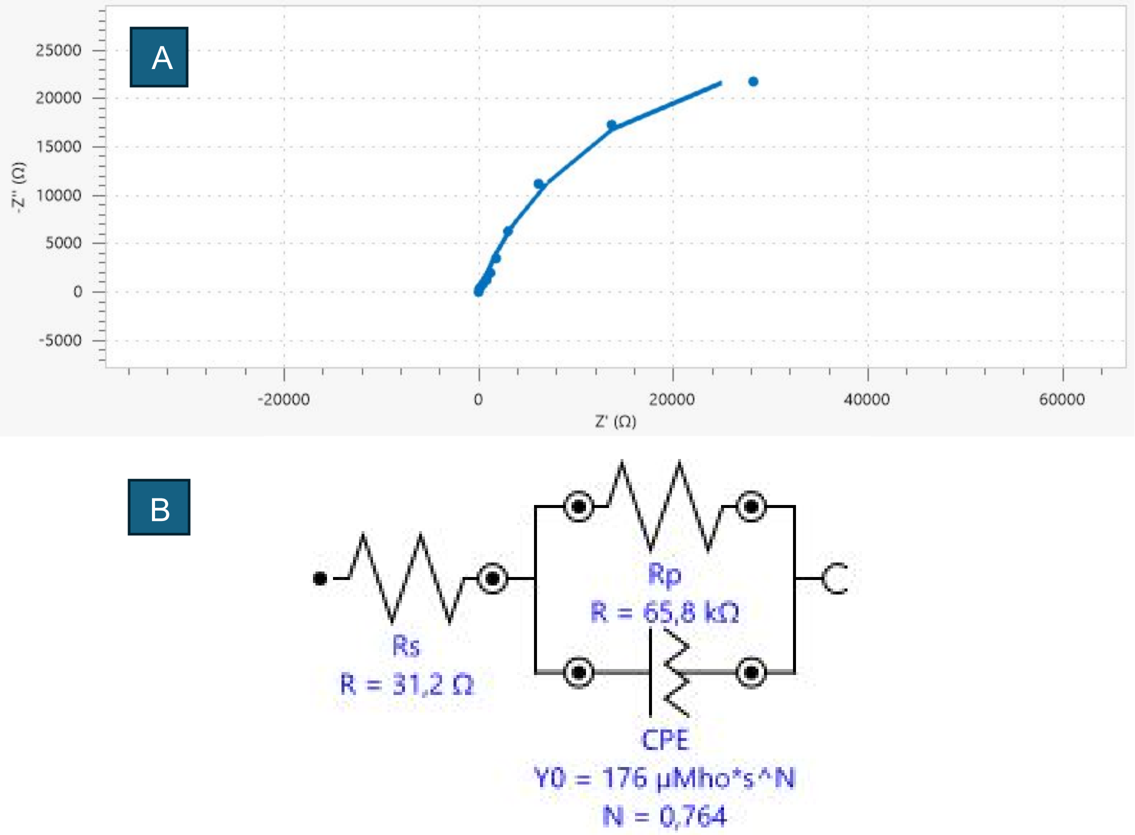

Figure 7A-B.SI Fitted EIS Diagram in A and equivalent circuit in B to TiO_2 film under FTO produced using Tween-80.
